# Can Integrated Care Help in Meeting the Challenges Posed on Our Health Care Systems by COVID-19? Some Preliminary Lessons Learned from the European VIGOUR Project

**DOI:** 10.5334/ijic.5596

**Published:** 2020-10-19

**Authors:** Sonja Lindner, Lutz Kubitschke, Christos Lionis, Marilena Anastasaki, Ursula Kirchmayer, Simona Giacomini, Vincenzo De Luca, Guido Iaccarino, Maddalena Illario, Antonio Maddalena, Antonio Maritati, Diego Conforti, Isabella Roba, Daniele Musian, Antonio Cano, Monica Granell, Ana M. Carriazo, Carmen M. Lama, Susana Rodríguez, Agnieszka Guligowska, Tomasz Kostka, Annemieke Konijnendijk, Maria Vitullo, Alejandro García-Rudolph, Javier Solana Sánchez, Marcello Maggio, Giuseppe Liotta, Chariklia Tziraki, Regina Roller-Wirnsberger, on behalf of the VIGOUR consortium

**Affiliations:** 1 Medical University of Graz, Department of Internal Medicine, Graz, AT; 2 Empirica GmbH, Bonn, DE; 3 Clinic of Social and Family Medicine, School of Medicine, University of Crete, GR; 4 Department of Epidemiology ASL Roma 1, Lazio Regional Health Service, IT; 5 ASL Viterbo, Lazio Regional Health Service, IT; 6 Research and Development Unit, Federico II University Hospital, Naples, IT; 7 Department of Advanced Biomedical Sciences, Federico II University and Hospital, Naples, IT; 8 Campania Region Health Innovation Unit, and Federico II University Department of Public Health, Naples, IT; 9 Local Health Agency Naples 1 Department for Home Care Services, Naples, IT; 10 ProMIS Coordination, Health Committee and Social & Health Relations Organization Unit Social and Health Area, Veneto Region, IT; 11 Department of Health and Social Policies, Autonomous Province of Trento, IT; 12 ALISA Liguria Region, Genoa, IT; 13 Department of Pediatrics, Obstetrics and Gynecology – INCLIVA, University of Valencia, Valencia, ES; 14 Department of Pediatrics, Obstetrics and Gynecology, University of Valencia, Valencia, ES; 15 Regional Ministry of Health and Families of Andalusia, Seville, ES; 16 Department of Geriatrics, Healthy Ageing Research Centre, Medical University of Lodz, PL; 17 University of Twente, Department of Biomedical Signals and Systems, Enschede, NL; 18 Santobono-Pausilipon Hospital, Naples, IT; 19 Department of Research and Innovation, Institut Guttmann, Institut Universitari de Neurorehabilitació adscrit a la UAB, Badalona, Barcelona, ES; 20 Universitat Autònoma de Barcelona, Bellaterra (Cerdanyola del Vallès), ES; 21 Fundació Institut d’Investigació en Ciències de la Salut Germans Trias i Pujol, Badalona, Barcelona, ES; 22 University of Parma, Department of Medicine and Surgery, IT; 23 University of Rome Tor Vergata, Department of Biomedicine and Prevention, Rome, IT

**Keywords:** integrated care, COVID-19, pandemic management, vulnerable patients, health care, social care

## Abstract

The COVID-19 pandemic puts health and care systems under pressure globally. This current paper highlights challenges arising in the care for older and vulnerable populations in this context and reflects upon possible perspectives for different systems making use of nested integrated care approaches adapted during the work of the EU-funded project VIGOUR (“Evidence based Guidance to Scale-up Integrated Care in Europe”, funded by the European Union’s Health Programme 2014–2020 under Grant Agreement Number 826640).

## Context and aim

The spread of the new coronavirus SARS-CoV-2 is challenging many health and care systems around the globe [1]. Its onset constitutes a critical issue, especially for older and/or vulnerable people and patients with pre-existing medical conditions being at risk for severe outcomes [2]. This new challenge puts care systems in need of answers: how can vulnerable populations be protected from becoming infected while also getting the best care in this exceptional situation, also considering that the attribution of vulnerability may need to be redefined within this pandemic [3]. In addition, how can care systems be equipped to continue providing complex care management in times of social isolation and containment?

Prior to the outbreak, care authorities from six European countries have joined forces in the VIGOUR project funded under the 3^rd^ European Health Programme in order to systematically review current practices in the health and care sector to see how existing services could be improved [4]. Taking current service delivery processes as point of departure, participating health care authorities had started to systematically analyse how current processes can be scaled-up to deliver better joined-up care. However, the pandemic has gradually reached all VIGOUR countries since the beginning of this year. The aim of this perspective paper is to draw lessons from the VIGOUR partners’ COVID-19 experience to date with regard to improving current practices by means of better integrating service delivery across health and social care.

## Integrated care for older and vulnerable citizens in Europe and the VIGOUR project

Integration of health and social care is widely advocated as way to improve the management and outcomes for increasing numbers of older, vulnerable people with varying and/or complex health and social care needs [5,6] with the goal to improve quality of care, quality of life, patient satisfaction and efficiency of care provision [7]. However, the implementation of structural changes in care delivery has often proved difficult in everyday practice [8,9]. One aspect adding complexity in this respect concerns the fact that integrated care represents a “nested” concept rather than a pre-defined organisational model of care delivery [10]. In practice, integrated care is strongly context bound, can take different forms [7,11] and there is a strong processual element in its implementation, e.g. when it comes to enabling cooperation and coordination processes involving different parties across care settings [8,12]. Such processes can take different forms depending on the given care contexts [12,13,14,15].

**Figure 1:**
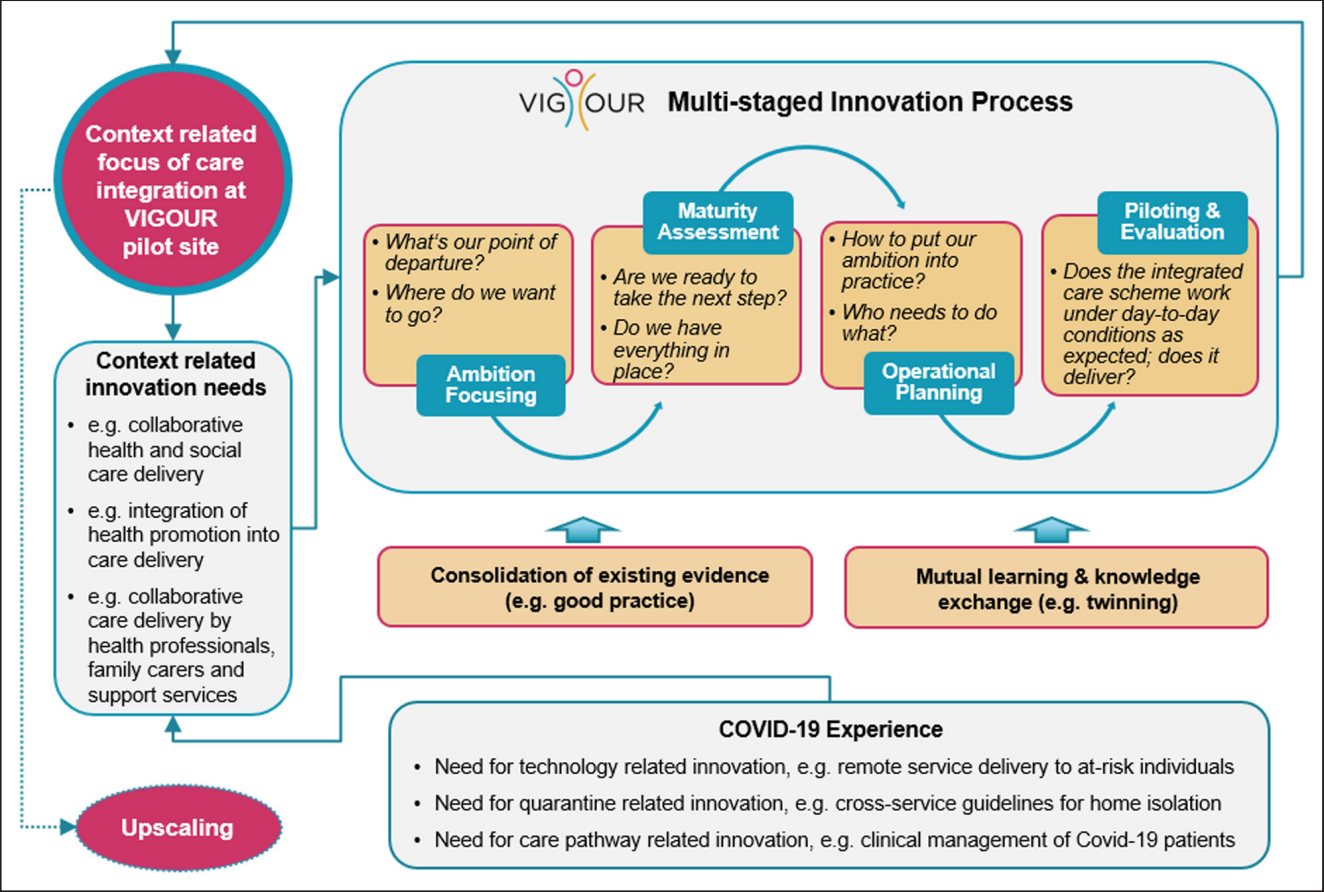
The VIGOUR Process. **Legend Figure 1:** Figure 1 demonstrates the multi-staged innovation process of the VIGOUR project, accompanying measures such as the consolidation of good practices and twinning actions and how experiences with COVID-19 affect the context-related innovation needs for each pilot site.

Against this background, a multi-staged process was developed by the VIGOUR project [16] to support participating stakeholders in identifying and implementing innovative practices with a view to better joining up hitherto separated care delivery processes (Figure 1). Each care authority can build on previous efforts to better align care delivery across the care chain, albeit in different ways and to different degrees. The VIGOUR process therefore begins with a targeted consolidation of the integration ambition, which is to be pursued by each care authority throughout the project. This is followed by a systematic assessment of the desired integration approach with respect to its appropriateness and feasibility under given framework conditions. Next, an operational implementation plan is developed as basis for piloting and evaluating the newly developed integration approach under day-to-day conditions with a view to preparing further upscaling. This process is further supported by means of knowledge transfer and mutual learning.

## Acute crisis and its implications on integrated care

The experiences gained during the pandemic so far reinforce the care integration approach of the VIGOUR project and encourage to build more connected health and care systems enabling collaboration across care settings and disciplines [17,18]. Effective responses to the COVID-19 pandemic require quick, collaborative and large-scale reactions; however, the current fragmentation in health and care systems inhibits these requirements. Maybe the present circumstances allow us to perceive the pandemic as a catalyst to redesign and integrate care pathways, also equipping us for any disruptive changes that may come beyond COVID-19 [17].

VIGOUR care authorities detected features gaining a higher importance during the pandemic outbreak, evolving into three major requirements to drive innovation in integrated care under emergency situations within health and care systems. First, the increased need for technology related innovation with regard to COVID-19 was recognized. Major aspects that gained momentum were triage and (remote) pre-triage, tele-consulting and tele-monitoring of COVID-19 positive patients and suspected cases, employment of screening or mobile diagnostic applications, contact tracing and monitoring of hospital and ICU beds availabilities. Second, VIGOUR pilot sites detected the requirement for quarantine related innovation during the pandemic outbreak. Social isolation and quarantine are required to be managed by means of cross-service guidelines for home isolation, by fostering at-home physical activity during quarantine and keeping remote contact with lifestyle coaches. Third, dynamics were also facilitated in the field of care pathway related innovation. A stronger involvement of case managers, personalized care planning efforts for COVID-19 patients, the development of dedicated integrated care processes and clinical pathways for patients and suspected cases and an enhancement of advanced care planning in long-term care represent underlying mechanisms anticipated.

Figure 1 illustrates the project approach and how preliminary experiences with COVID-19 were incorporated into the process.

Reflecting upon the VIGOUR progress so far, some preliminary lessons learned can be drawn on how envisaged care integration approaches of the pilot sites were shaped within the course of the pandemic.

Not surprisingly, eHealth and digitalization in their various characteristics represent a valuable tool for facilitating integrated care processes also during the COVID-19 pandemic. The COVID-19 experiences that the VIGOUR partners have been able to make so far have made particularly clear the potential generally provided by digital technologies for the provision of person-centred and coordinated integrated care. However, the availability of practical and safe applications is crucial [19]. This fact has also been highlighted recently in a report released by the International Foundation of Integrated Care (IFIC) [17]. Still, it would be a false inverse conclusion to expect that digital technologies automatically lead to better care [20]. Indeed, usability and benefit of digital technologies in integrated care strongly depend on the context and needs of the target populations.

Social isolation was discovered as hotspot, as on the one hand, it has proven to be a necessity to avoid transmission of the COVID-19 infection and on the other hand, isolation may lead to deeper psychological and mental health issues, especially for older, vulnerable citizens [21,22]. Literature highlights that isolation or loneliness has a detrimental effect on health, with depression and cardiovascular health as outcomes most researched [23]. The introduction of dedicated clinical pathways, integrated care process management for COVID-19 patients and case managers are seen as reasonable healthcare practices by VIGOUR partners helping to maintain healthcare capacities and guarantee integrated care provision in pandemic times. Especially the employment of case managers and care coordinators helps to overcome fragmented healthcare organization. This fact is getting even more relevant when considering the still existing underrepresentation of case management and care coordination in integrated care for the management of an ageing population [24] whereas integrating primary care with hospital care enables the establishment of a care continuum for patients [25].

Additionally, the role of primary health care in regards to the development of an integrated care system has received prompt attention by VIGOUR partners and is on line with the World Health Organization (WHO) Anniversary Meeting in Astana [26].

The pandemic has brought to fragmentation and gaps in our social and health care systems and has accelerated the need for integration and coordination of health and social care. In order to achieve better integration, a realistic perspective is moving forward given the complexity and variety of culture and socio-political dependant variables. The framework within VIGOUR project partners takes into consideration the fact “that one model does not fit all”. Thus, the insights and various models developed during the project will assist the exploration, development and implementation of different care integration approaches in distinct systems across Europe [27] as well as in times of the pandemic and beyond [18].

## Conclusion

The onset of COVID-19 constitutes a critical issue and forces health care systems not to just provide acute care opportunities for COVID-19 patients, but also to rethink and redesign care pathways. The VIGOUR project approach seems robust to influences evoked by the pandemic and flexible enough to take advantage of integrated care initiatives available on pilot level and adopt them to specific needs emerging in pandemic times. eHealth, quarantine management and integrated clinical management of COVID-19 patients and suspected cases evolved into promising aspects, leading health and social care systems towards a more integrated care approach. Further information on this topic may be expected from VIGOUR by fall 2021.
